# Proton-pump inhibitor vs. H2-receptor blocker use and overall risk of CKD progression

**DOI:** 10.1186/s12882-021-02449-0

**Published:** 2021-07-15

**Authors:** Liza Cholin, Tarek Ashour, Ali Mehdi, Jonathan J. Taliercio, Remy Daou, Susana Arrigain, Jesse D. Schold, George Thomas, Joseph Nally, Nazih L. Nakhoul, Georges N. Nakhoul

**Affiliations:** 1 Cleveland Clinic, Cleveland, OH USA; 2 Saint-Joseph University, Beirut, Lebanon; 3 Tulane University School of Medicine, New Orleans, LA USA

**Keywords:** Proton-pump inhibitors, Histamine-2 receptor blockers, Chronic kidney disease, Disease progression, Mortality

## Abstract

**Background:**

The relationship between proton-pump inhibitor (PPI) use and chronic kidney disease (CKD) progression remains controversial. Specifically, there is a lack of data evaluating renal outcomes in established CKD patients. The aim of our study is to determine the risk of progression to end-stage kidney disease (ESKD) or death amongst CKD patients on PPI, histamine-2 receptor blocker (H2B), or no anti-acid therapy.

**Methods:**

Using our CKD registry, we evaluated the relationship between PPI and H2B use and outcomes amongst patients with CKD (eGFR < 60), with at least 2 PCP visits in the year prior. A Cox proportional hazards model was used to evaluate the relationship between medication groups and overall mortality, while competing risks regression models were used to determine the risk of ESKD with death as a competing risk.

**Results:**

25,455 patients met inclusion criteria and were stratified according to medication group: no antacid therapy (15,961), PPI use (8646), or H2B use (848). At 4 years, the cumulative incidence of ESKD with death as a competing risk was 2.0% (95% CI: 1.7, 2.4), 1.5% (0.8, 2.8), and 1.6%(1.4, 1.9) among PPI, H2B, and no medication respectively (*P* = 0.22). The cumulative incidence of death with ESKD as a competing risk was 17.6% (95% CI: 16.6, 18.6), 16.7% (13.7, 19.8), and 17.3% (16.6, 18.0) (*P* = 0.71).

**Conclusions:**

Use of PPI in a CKD population was not associated with increased mortality or progression to ESKD when compared to H2 blocker and to no acid suppressing therapy.

**Supplementary Information:**

The online version contains supplementary material available at 10.1186/s12882-021-02449-0.

## Introduction

Since the introduction of Omeprazole in 1989, Proton Pump Inhibitors (PPIs) have gone on to become one of the most widely prescribed medications in the United States. These drugs are also increasingly sold over the counter (OTC), making the actual use of PPIs across the general population much higher. Initially praised for their superiority in treating many acid-related disorders, PPIs have more recently garnered scrutiny over their potential adverse effects [1–5]. Acute kidney injury (AKI) associated with PPI use is well documented in the literature. Case reports and small case series initially described the development of acute interstitial nephritis (AIN) in patients prescribed PPIs [6, 7]. Since that time, there have been various large observational studies and meta-analyses which have reproduced the same findings of increased risk of developing AKI with PPI use [3–5, 8, 9].

More recently, some studies have suggested an increased risk of developing chronic kidney disease (CKD) among patients with normal renal function [10–14]. The underlying mechanism of this association remains unclear. A popular theory that an intervening AKI in the setting of PPI use leads to the development of CKD was not upheld when examined by Xie and colleagues [15]. A recent systematic review and meta-analysis also demonstrated a higher incidence of CKD with PPI use [9]. Interestingly, the subgroup analysis found no association between PPI use and risk of CKD in older patients (age > 62 years), in studies with large sample sizes (> 10,000 participants), in case-control studies, and in the US study location (3 out of the 4 studies reviewed) [9]. Despite the growing body of evidence on PPI use and increased risk for developing CKD, there is limited data on patients with already established CKD. To the best of our knowledge, only one such study has been published [16]. It concluded that PPI use was associated with increased major renal adverse events, defined as doubling of serum creatinine or progression to end-stage renal disease.

Given the inconclusive data on PPI safety in the CKD population, we elected to examine the rate of CKD progression among CKD patients on PPI versus Histamine-2-Receptor Blockers (H2B) and to no acid-suppressing therapy.

## Materials and methods

### Patient population

We used data from our Electronic Health Record (EHR) - based Chronic Kidney Disease (CKD) registry to evaluate the relationship between the use of proton-pump inhibitors (PPI) and H2 blockers and outcomes. We have previously described the development and validation of this registry [16]. For the current analysis, we included patients who: a) had at least one face-to-face outpatient encounter with a Cleveland Clinic health care provider and at least two estimated glomerular filtration rate (eGFR) measures < 60 ml/min/1.73 m^2^, > 90 days apart, between January 1, 2007 and December 30, 2017 and patients who were not on dialysis nor had a functioning kidney transplant, b) had active PPI and no prior H2B prescription, or had active H2B and no prior PPI prescription, or had neither prior PPI nor prior H2B on the date of second eGFR< 60 (CKD), c) were residents of the State of Ohio, and d) had continuity of care at our institution with at least 2 PCP visits in the year prior to the second eGFR< 60.

### Patient characteristics

We extracted demographic information from the EHR, and defined comorbid conditions such as diabetes mellitus, hypertension, coronary artery disease, malignancy, congestive heart failure, and hyperlipidemia using pre-specified and previously validated criteria [17]. We evaluated whether these conditions existed prior to the second eGFR < 60 ml/min/1.73 m^2^ which was the inception date for each patient. We also extracted relevant laboratory data (serum albumin, potassium and bicarbonate, proteinuria) from the EHR. For laboratory results, the last outpatient laboratory result obtained within 2 years prior to inception was included.

We evaluated whether patients had continuity of care at our institution by searching for 2 completed visits or appointments with the listed primary care provider (PCP) within the year prior to the second eGFR < 60 ml/min/1.73 m^2^.

### Kidney function

All serum creatinine measurements were done in the same laboratory using a Hitachi D 2400 Modular Chemistry Analyzer (Roche Diagnostics, Indianapolis, IN). We estimated eGFR using the CKD-EPI equation [18]. CKD was classified into the following stages: stage 3 CKD (eGFR 30–59 ml/min/1.73 m^2^), stage 4 CKD (eGFR 15–29 ml/min/1.73 m^2^), and stage 5 CKD (eGFR < 15 ml/min/1.73 m^2^). We further categorized stage 3 into CKD stage 3a (eGFR 45–59 ml/min/1.73 m^2^) and stage 3b (eGFR 30–44 ml/min/1.73 m^2^).

### Proton pump inhibitors and H2 blockers

Prescriptions for PPI and H2B were obtained for all patients from the EHR. The prescriptions were written by the medical providers, and we could not verify with area pharmacies whether the prescriptions were filled. When a prescription had a start date before the second eGFR< 60 and an end date after that second eGFR< 60, the prescription was considered active at baseline and the patient was grouped accordingly. All PPI and H2B prescriptions as described in the previous sentence were considered active regardless of duration or dose. Patients were grouped into mutually exclusive groups on the date of second eGFR< 60: a) active PPI and no record of prior H2B, b) active H2B and no prior PPI, or c) no prior PPI or H2B.

### Outcomes

Our outcomes were mortality, end-stage kidney disease (ESKD) with death as a competing risk, and death with ESKD as a competing risk. We ascertained dates of death from the EHR as well as through linkage of the CKD registry with the Ohio Department of Health death records. We ascertained ESKD through linkage of our CKD registry with the United States Renal Data Services (USRDS). The end points of ESKD and transplant were derived from the United States Renal Data System (USRDS). The USRDS is funded by the National Institute of Diabetes and Digestive Kidney Diseases and is a national data registry that collects information including treatment and outcomes on the ESKD population in the US [19]. In our study, ESKD was defined as either initiation of long-term dialysis or pre-emptive transplant. Deaths and ESKD were obtained up to end of 2017.

### Statistical analysis

We compared baseline characteristics among patients with PPI, H2B and no medication using Chi-square, ANOVA and Kruskal-Wallis tests for categorical and continuous variables, respectively. The inception date for each patient was the date of 2nd eGFR< 60. We estimated Kaplan-Meier survival over time by medication group and also the cumulative incidence function of ESKD with death as a competing risk, and the cumulative incidence function of death with ESKD as a competing risk. We compared the cumulative incidences across medication groups using Gray’s test for equality of cumulative incidence functions [20]. For overall survival, patients were followed from their inception dates until their death, or when death was unobserved, until the censoring date of 12/31/2017 (for overall survival patients were not censored at ESKD). For the competing risks analyses, the censoring date was also 12/31/2017.

We evaluated the relationship between medication group and overall mortality using a Cox proportional hazards models and the relationship between medication group and progression to ESKD using competing risks regression models as described by Fine and Gray [21]. We adjusted the models for the following covariates: age, race, sex, eGFR, BMI group, hemoglobin, potassium, CO2, diabetes, hypertension, CVD, PVD,CAD, CHF, malignancy, ACEI/ARB, beta blockers, smoking and insurance. We used natural cubic splines with 3 equally spaced knots (at the 25th, 50th and 75th percentiles) to relax linearity assumptions for continuous covariates when appropriate. We tested an interaction between CKD stage and PPI vs. no medication while excluding H2B patients. We excluded H2B patients because there were not many in advanced stages of CKD. The model testing the CKD stage vs. PPI/no medication interaction was adjusted for the same variables mentioned above except eGFR which was substituted for CKD stage in this model.

We had missing data for the following percent of patients: 1% missing BMI and smoking, 16% hemoglobin, 0.2% potassium and bicarbonate, and 3% insurance data. We used multiple imputations (SAS proc. MI) with the Markov Chain Monte Carlo method and a single chain to impute 5 datasets with complete continuous and binary data in a first step, and then in a second step we imputed insurance group on each of the 5 datasets using discriminant function analysis. All the covariates from the multivariable model were included in the imputation. We fit the models on each of the 5 imputed datasets, and parameter estimates were combined using SAS MI analyze.

We had proteinuria data on 13,157 (52%) of the patients in the study, and as a sensitivity analysis, we evaluated the adjusted mortality and competing risks models while adjusting for proteinuria data in this subset of patients.

All analyses were conducted using Linux SAS version 9.4 (SAS Institute, Cary, NC), and graphs were created using R 3.5.1 (The R Foundation for Statistical Computing, Vienna, Austria). This study and the CKD registry were both approved by the Cleveland Clinic Institutional Review Board.

## Results

**Fig. 1:**
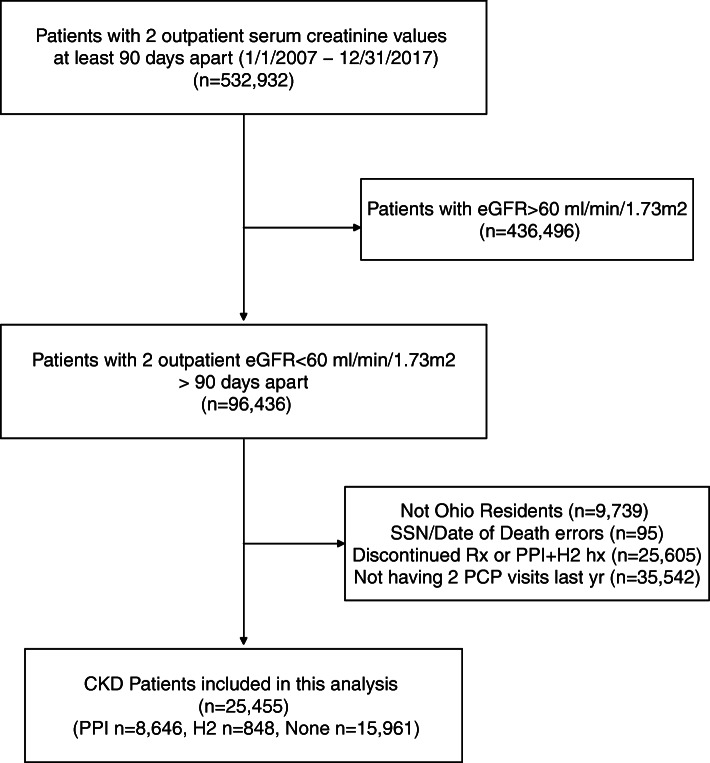
Flow chart of selection criteria

Out of 96,436 patients that entered the CKD registry between 2007 and 2017, there were 86,697 that were residents of Ohio (Fig. 1). Of those, 25,455 had 2 PCP visits in the year prior and fulfilled all inclusion criteria. Among them, 8646 had active PPI with no prior H2B, 848 had active H2B with no prior PPI, and 15,961 had neither medication. Patients on the different medication groups were significantly different on several characteristics (Table 1). Most notably, patients on PPI and H2B were less likely to be male, more likely to be obese and more likely to be on Medicare. Those on PPI were also more likely to have CAD and CHF.

**Table 1:** Patient characteristics by medication group

Factor	N missing	Overall (N = 25,455)	None (***N*** = 15,961)	PPI (***N*** = 8646)	H2 (***N*** = 848)	***p***-value
Age	0	73.2 ± 11.0	73.4 ± 11.1	72.8 ± 10.9	73.2 ± 11.4	0.001^a^
Male Sex	0	10,722 (42.1)	7092 (44.4)	3322 (38.4)	308 (36.3)	< 0.001^c^
African American	0	2982 (11.7)	1896 (11.9)	970 (11.2)	116 (13.7)	0.060^c^
Smoke	0					< 0.001^c^
No		23,190 (91.1)	14,486 (90.8)	7938 (91.8)	766 (90.3)	
Yes		2035 (8.0)	1300 (8.1)	663 (7.7)	72 (8.5)	
Missing		230 (0.90)	175 (1.1)	45 (0.52)	10 (1.2)	
BMI	270	30.1 ± 6.7	29.8 ± 6.6	30.5 ± 6.7	30.7 ± 7.3	< 0.001^a^
BMI group	0					< 0.001^c^
< 18.5 kg/m2		295 (1.2)	204 (1.3)	82 (0.95)	9 (1.1)	
18.5–24.9 kg/m2		5203 (20.4)	3475 (21.8)	1551 (17.9)	177 (20.9)	
25–29.9 kg/m2		8638 (33.9)	5446 (34.1)	2941 (34.0)	251 (29.6)	
30–34.9 kg/m2		6064 (23.8)	3654 (22.9)	2207 (25.5)	203 (23.9)	
35–39.9 kg/m2		2872 (11.3)	1703 (10.7)	1051 (12.2)	118 (13.9)	
40+ kg/m2		2113 (8.3)	1275 (8.0)	760 (8.8)	78 (9.2)	
Missing		270 (1.1)	204 (1.3)	54 (0.62)	12 (1.4)	
eGFR	0	50.0 ± 9.2	50.1 ± 9.1	49.8 ± 9.5	49.2 ± 9.0	0.004^a^
CKD stage	0					0.021^c^
3a. 45–59		19,584 (76.9)	12,333 (77.3)	6619 (76.6)	632 (74.5)	
3b. 30–44		4703 (18.5)	2940 (18.4)	1582 (18.3)	181 (21.3)	
4. 15–29		1054 (4.1)	624 (3.9)	400 (4.6)	30 (3.5)	
5. < 15		114 (0.45)	64 (0.40)	45 (0.52)	5 (0.59)	
Diabetes	0	8695 (34.2)	5276 (33.1)	3108 (35.9)	311 (36.7)	< 0.001^c^
Malignancy	0	5889 (23.1)	3646 (22.8)	2084 (24.1)	159 (18.8)	< 0.001^c^
Hypertension	0	24,775 (97.3)	15,487 (97.0)	8462 (97.9)	826 (97.4)	< 0.001^c^
Hyperlipidemia	0	22,525 (88.5)	14,012 (87.8)	7764 (89.8)	749 (88.3)	< 0.001^c^
Coronary artery disease	0	5340 (21.0)	2910 (18.2)	2243 (25.9)	187 (22.1)	< 0.001^c^
Congestive heart failure	0	2194 (8.6)	1175 (7.4)	941 (10.9)	78 (9.2)	< 0.001^c^
Cerebrovascular Disease	0	2974 (11.7)	1692 (10.6)	1170 (13.5)	112 (13.2)	< 0.001^c^
PVD	0	1387 (5.4)	786 (4.9)	555 (6.4)	46 (5.4)	< 0.001^c^
ACE/ARB	0	18,403 (72.3)	11,445 (71.7)	6354 (73.5)	604 (71.2)	0.009^c^
Diuretics	0	18,029 (70.8)	11,071 (69.4)	6354 (73.5)	604 (71.2)	< 0.001^c^
Statin	0	17,385 (68.3)	10,498 (65.8)	6302 (72.9)	585 (69.0)	< 0.001^c^
Beta Blockers	0	14,467 (56.8)	8508 (53.3)	5433 (62.8)	526 (62.0)	< 0.001^c^
Albumin	3359	4.1 ± 0.36	4.2 ± 0.35	4.1 ± 0.38	4.1 ± 0.35	< 0.001^a^
Hemoglobin	4019	13.1 ± 1.7	13.3 ± 1.7	12.8 ± 1.7	13.0 ± 1.7	< 0.001^a^
Proteinuria	12,298	3190 (24.2)	2013 (25.1)	1075 (22.9)	102 (23.0)	0.014^c^
CO2	64	25.8 ± 3.0	25.8 ± 3.0	25.7 ± 3.2	25.8 ± 3.1	0.007^a^
Potassium	51	4.4 ± 0.50	4.4 ± 0.49	4.4 ± 0.51	4.4 ± 0.51	0.36^a^
Urine ph	14,759	6.0 [5.0,6.5]	6.0 [5.0,6.5]	6.0 [5.0,6.5]	6.0 [5.0,6.0]	0.57^b^
Insurance grouped	0					< 0.001^c^
Medicaid		434 (1.7)	229 (1.4)	189 (2.2)	16 (1.9)	
Medicare		18,508 (72.7)	11,397 (71.4)	6463 (74.8)	648 (76.4)	
Missing		760 (3.0)	492 (3.1)	249 (2.9)	19 (2.2)	
Other		5753 (22.6)	3843 (24.1)	1745 (20.2)	165 (19.5)	

Statistics presented as Mean ± SD, Median [P25, P75] or N (column %)

*p*-values: ^a^ = ANOVA, ^b^ = Kruskal-Wallis test, ^c^ = Pearson’s chi-square test

### Mortality

During a median follow up of 4.14 years, there were 5562 deaths, 196 in the H2B group, 1845 in the PPI group, and 3521 in the no medication group. At 1-year the Kaplan-Meier all-cause mortality survival estimates were 96.1(95.8, 96.4), 96.3 (95.9, 96.7) and 98.0 (97.0, 99.0) for the no medication, PPI and H2B groups respectively. At 4 years, survival was 82.2 (81.5, 82.9), 81.8 (80.8, 82.8) and 83.0 (79.9, 86.1) respectively (Log-rank *P* = 0.56, Additional file 1: Figure 1). Table 2 shows results from the adjusted Cox mortality model. When adjusted for comorbidities and laboratory results, medication group was not significantly associated with hazard of death. The interaction between CKD stage and PPI vs. no medication (excluding H2B) was not significant (*P* = 0.96, *N* = 24,607) suggesting that the relationship between PPI and mortality is not different across different CKD stages.

**Table 2:** Adjusted models: Cox model of mortality and Competing risks Regression models of ESKD and Death

Medication group	Cox model of Mortality HR (95% CI)* (N = 25,455, death events = 5562)	P-value	Competing Risks ESKD with death as competing risk SHR (95% CI)* (N = 25,455, ESKD events = 452, death events = 5362)	P-value	Competing Risks Death with ESKD as competing risk SHR (95% CI)* (***N*** = 25,455, ESKD events = 452, death events = 5362)	***P***-value
PPI vs. none	0.97 (0.91, 1.03)	0.29	0.93 (0.75, 1.15)	0.49	0.96 (0.90, 1.02)	0.15
H2 vs. none	0.99 (0.86, 1.15)	0.90	1.07 (0.67, 1.73)	0.77	1.01 (0.87, 1.17)	0.95
PPI vs. H2	0.98 (0.84, 1.13)	0.77	0.87 (0.53, 1.42)	0.57	0.95 (0.82, 1.11)	0.53

*Adjusted for age, race, sex, eGFR, BMI group, hemoglobin, potassium, CO2, diabetes, hypertension, CVD, PVD,CAD, CHF, malignancy, ACE/ARB, beta blockers, smoking and insurance

All adjusted models used 5 datasets created with multiple imputation and MI analyze to obtain the HR or SHR

### Progression to ESKD with death as a competing risk

**Fig. 2:**
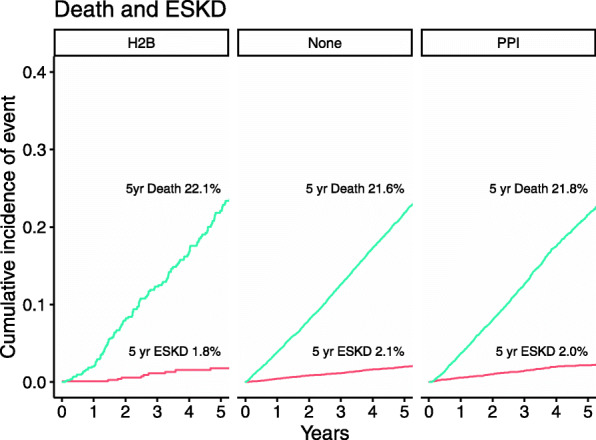
Risk of death and progression to ESKD by medication group

With a median follow up of 4.1 years, there were 452 ESKD events and 5362 deaths. In unadjusted analysis, the cumulative incidence of ESKD with death as a competing risk was not significantly different across groups (*P* = 0.22, Fig. 2). At 4 years, it was 2.0% among PPI (95% CI: 1.7, 2.4), 1.5% among H2B (95% CI: 0.8, 2.8), and 1.6% among those with neither medication (95% CI: 1.4, 1.9). The cumulative incidence of death with ESKD as a competing risk was also not significantly different across groups (*P* = 0.71). At 4 years, it was 17.6% among PPI (95% CI: 16.6, 18.6), 16.7% among H2B (95% CI: 13.7, 19.8), and 17.3% among those with neither medication (95% CI: 16.6, 18.0).

In an adjusted competing risks regression model, medication group was not significantly associated with ESKD while considering death as a competing risk (Table 2). Medication group was also not significantly associated with death while considering ESKD as a competing risk. The interaction between CKD stage and PPI vs. no medication group (excluding H2B) was not significant in the model of ESKD with death as a competing risk (*P* = 0.11, *N* = 24,607), or in the model of death with ESKD as a competing risk (*P* = 0.80, N = 24,607). The lack of interaction suggests the correlation between medication group and outcomes is not different across CKD stages.

In our sensitivity analysis adjusted for proteinuria (*N* = 13,157), we found that mortality was not significantly different across groups (PPI vs. none HR = 0.97, 95% CI: 0.89, 1.05, PPI vs. H2B HR = 1.03, 95% CI: 0.84, 1.28, H2B vs. none HR = 0.94, 95% CI: 0.76, 1.16). ESKD with death as a competing risk was also not significantly different across groups (PPI vs. none SHR = 0.93, 95% CI: 0.71, 1.22, PPI vs. H2B SHR = 0.86, 95%CI: 0.47, 1.61, and H2B vs. none SHR = 1.08, 95% CI: 0.60, 1.95), and death with ESKD as a competing risk was also not different across groups (PPI vs. none SHR = 0.95, 95% CI: 0.87, 1.04, PPI vs. H2B SHR = 1.02, 95%CI: 0.81, 1.28, and H2B vs. none SHR = 0.93, 95% CI: 0.74, 1.17).

## Discussion

Our study shows that in a population of established stage III to V CKD, there is no association between use of PPI, use of H2-Receptor Blockers and progression to ESKD. The study further determined that there is no difference between the medication groups in overall mortality.

Concerning the progression to ESKD, our results are in contrast to the findings of several other large cohort studies looking at incidence of CKD and rate of CKD progression among PPI users. Several important differences could explain why our findings were different. First, our study was specifically looking at patients with reduced eGFR at baseline (i.e. established CKD patients), while most other cohorts analyzed patients with normal baseline renal function [10–12, 14]. In the Klatte study, although patients with reduced renal function were not excluded, the median eGFR was 94.2 and 88.6 ml/min/m2 in the H2B and PPI groups, respectively [13]. The Xie study also had limitations that made interpreting the data difficult. In the earlier follow up period, the study suggested an increased risk of incident CKD and CKD progression; however, after 720 days, the risk of declining renal function actually started to decrease in the PPI group [12]. The Klatte and Xie studies were also later compiled in a meta-analysis review, which found no association between PPI use and adverse renal outcomes in the subgroup analysis section [9]. The study done by Grant and colleagues concluded that PPI use is associated with increased risk of major adverse renal events [16]. This study is the only other study, to date, to look at PPI safety specifically in the CKD population. However, there are several important distinctions between Grant’s study and our own, which could account for the differences seen in our results. First, there was a large portion of patients excluded from the original cohort (50%), which could have led to a selection bias. Second, the baseline characteristics were different amongst the two groups tested. The PPI group had more patients with lower eGFR, more proteinuria, and higher prevalence of myocardial infarction and diabetes. These characteristics are known CKD risk factors; therefore, some of the positive association with PPI use and adverse renal outcomes could actually be due to the PPI group being sicker at baseline.

Association between PPI use and all-cause mortality is difficult to interpret due to the large variance in patients studied to date. PPI use has been associated with increased mortality in cancer patients, liver cirrhotics, and those requiring artificial nutrition [22–24]. However, these patient cohorts are dissimilar to the CKD population, and thus of limited use in determining true risk. Of the studies that more closely resemble our patient cohort, there are some that also suggest increased mortality in patients using PPIs. Xie and colleagues showed a small excess of cause specific mortality due to cardiovascular disease, chronic kidney, disease, and upper gastrointestinal cancer in patients taking PPIs [25]. In addition, another cohort study determined increased risk of overall mortality with PPI use in kidney transplant recipients with average eGFR of 52 [26]. These findings are in contrast with the results from Grant’s study which looked at the CKD population specifically and found no association between PPI use and increased mortality. Overall, the reports to date have major limitations including the observational nature of the studies and the degree of illness in the cohorts analyzed. Our study could find no association between PPI use and overall mortality when assessing patients with pre-established CKD.

Strengths of this analysis include a diverse population of stage III-V CKD, availability of several confounding factors, and the inclusion of patients with at least 2 PCP visits. By only including patients with at least 2 PCP visits, we ensured continuity of care, medication reconciliation and verification of continued acid-suppression therapy use, and avoided a potential selection bias from loss to follow up. As patients in the PPI group were sicker at baseline, the lack of association between PPI use and increased risk of adverse renal outcomes is less likely to have been influenced by confounding variables.

Our study had several limitations as well. Similar to other observational studies, we were not able to confirm medication compliance or whether or not there was additional PPI use over the counter. The cumulative dose and duration of prescribed PPI could also not be established. Due to this fact, it is possible that patients that were on PPI for more extended periods of time might have been impacted by the medication differently than on-demand users. However, by requiring 2 PCP visits, we were able to partially mitigate this issue by ensuring PPI use was documented at two different points in the study. Incident PPI users were, therefore, not included in the final patient cohort. Another limitation was the retrospective nature of the study, which made it prone to residual confounding. While we included several variables that could affect mortality, we lacked details about nutritional data and albuminuria. Further, our patients have been followed in a health care system and hence these data might not be applicable to the community-dwelling adults with CKD. Finally, though including at least 2 PCP visits as an inclusion criterion helped minimize selection bias, it also reduced our sample size; therefore potentially making our findings less significant.

In summary, the use of PPI in a CKD population was not associated with increased risk of CKD progression compared to the use of H2B and to no acid-suppression therapy. More prospective research is needed to solidify those results and to identify the safety of PPI in CKD populations.

## Conclusion

Similar rates of progression to ESKD and overall mortality were noted among PPI vs H2B users in the CKD population. There were several limitations to this study, the biggest one being the inability to confirm duration of prescription use. However, the results from this study are still relevant, as a large portion of the CKD population rely on acid-suppressing therapy to manage their co-morbidities. Although more prospective research is still needed, the results from this study suggest that proton-pump inhibitors may be safe for use in select CKD patients.

## Supplementary Information

**Additional file 1.**

## Data Availability

All data analyzed during this study are included in this published article.
