# Supplementary material for: Proton-pump inhibitor vs. H2-receptor blocker use and overall risk of CKD progression
Source: BMC Nephrol. 2021 Jul 15;22:264. doi: 10.1186/s12882-021-02449-0 (PMC8281649; doi:10.1186/s12882-021-02449-0)
Supplement: Supplementary file 1 — Additional file 1. [file 12882_2021_2449_MOESM1_ESM.docx]

|  | **Cox model of Mortality**  **HR (95% CI)**  **(N=25,455, death events=5,562)** | **P-value** | **Competing Risks ESKD with death as competing risk**  **SHR (95% CI)**  **(N=25,455, ESKD events=452, death events=5,362)** | **P-value** | **Competing Risks Death with ESKD as competing risk**  **SHR (95% CI)**  **(N=25,455, ESKD events=452, death events=5,362)** | **P-value** |
| --- | --- | --- | --- | --- | --- | --- |
| Age with spline term 1 | * | <0.001 | * | <0.001 | * | <0.001 |
| Age with spline term 2 | * | <0.001 | * | 0.006 | * | <0.001 |
| Male sex | 1.41 (1.33, 1.49) | <0.001 | 2.02 (1.64, 2.49) | <0.001 | 1.38 (1.29, 1.46) | <0.001 |
| African American vs. not | 0.85 (0.78, 0.93) | <0.001 | 1.56 (1.24, 1.95) | <0.001 | 0.83 (0.75, 0.91) | <0.001 |
| Smoke vs. not | 1.62 (1.47, 1.79) | <0.001 | 1.02 (0.76, 1.36) | 0.91 | 1.63 (1.46, 1.81) | <0.001 |
| *BMI group* |  |  |  |  |  |  |
| <18.5 kg/m2 | 1.46 (1.21, 1.76) | <0.001 | 1.68 (0.72, 3.94) | 0.23 | 1.44 (1.15, 1.81) | 0.002 |
| 18.5-24.9 kg/m2 | Ref |  | Ref |  | Ref |  |
| 25-29.9 kg/m2 | 0.78 (0.73, 0.84) | <0.001 | 0.93 (0.67, 1.29) | 0.67 | 0.79 (0.73, 0.85) | <0.001 |
| 30+ kg/m2 | 0.76 (0.71, 0.82) | <0.001 | 0.89 (0.65, 1.21) | 0.46 | 0.77 (0.72, 0.83) | <0.001 |
| eGFR per 1 unit higher | 0.98 (0.989, 0.985) | <0.001 | 0.90 (0.89, 0.91) | <0.001 | 0.99 (0.986, 0.992) | <0.001 |
| Diabetes | 1.29 (1.21, 1.37) | <0.001 | 1.92 (1.55, 2.38) | <0.001 | 1.24 (1.17, 1.33) | <0.001 |
| Malignancy | 1.35 (1.27, 1.43) | <0.001 | 0.59 (0.43, 0.81) | 0.001 | 1.36 (1.28, 1.45) | <0.001 |
| Hypertension | 0.86 (0.74, 1.005) | 0.06 | 1.06 (0.47, 2.38) | 0.89 | 0.86 (0.74, 0.99) | 0.039 |
| Coronary Artery Disease | 1.15 (1.08, 1.23) | <0.001 | 1.11 (0.86, 1.43) | 0.41 | 1.15 (1.07, 1.23) | <0.001 |
| Congestive Heart Failure | 2.01 (1.86, 2.18) | <0.001 | 1.18 (0.90, 1.55) | 0.22 | 1.97 (1.80, 2.15) | <0.001 |
| Cerebrovascular Disease | 1.24 (1.15, 1.33) | <0.001 | 0.89 (0.65, 1.20) | 0.43 | 1.24 (1.15, 1.34) | <0.001 |
| PVD | 1.37 (1.23, 1.52) | <0.001 | 1.26 (0.88, 1.80) | 0.21 | 1.32 (1.18, 1.47) | <0.001 |
| ACE/ARB (history of) | 0.89 (0.84, 0.95) | <0.001 | 1.99 (1.43, 2.77) | <0.001 | 0.88 (0.83, 0.94) | <0.001 |
| Beta Blockers (history of) | 1.17 (1.10, 1.24) | <0.001 | 1.43 (1.13, 1.82) | 0.003 | 1.16 (1.09, 1.23) | <0.001 |
| CO2 with spline term 1 | * | <0.001 | * | 0.91 | * | <0.001 |
| CO2 with spline term 2 | * | <0.001 | * | 0.26 | * | <0.001 |
| Hemoglobin with spline term 1 | * | <0.001 | * | 0.046 | * | <0.001 |
| Hemoglobin with spline term 2 | * | <0.001 | * | 0.039 | * | <0.001 |
| Medicare/Medicaid vs. others | 0.87 (0.81, 0.93) | <0.001 | 1.59 (1.22, 2.08) | <0.001 | 0.86 (0.80, 0.92) | <0.001 |
| Potassium with spline term 1 | * | 0.016 | * | 0.49 | * | 0.0496 |
| Potassium with spline term 2 | * | 0.017 | * | 0.68 | * | 0.039 |

*Parameter cannot be interpreted due to spline
